# Design and Rationale of the Safe Surveillance of PCI Under Mechanical Circulatory Support With the Saranas Early Bird Bleed Monitoring System (SAFE-MCS) Study

**DOI:** 10.1016/j.jscai.2023.101049

**Published:** 2023-07-05

**Authors:** Philippe Généreux, Amir Kaki, Mostafa Naguib, Brittany Fuller, Hursh Naik, Michael Kim, Amirali Masoumi, Thomas Waggoner, Zaffer Syed, Julia Walsh, Dimitri Karmpaliotis, Mir Babar Basir

**Affiliations:** a Gagnon Cardiovascular Institute, Morristown Medical Center, Morristown, New Jersey; b Ascension St. John Hospital, Detroit, Michigan; c Henry Ford Health System, Detroit, Michigan; d St. Joseph’s Medical Center, Phoenix, Arizona; e Lenox Hill Hospital, Northwell Health, New York, New York; f USHV Heart & Vascular, Tucson Medical Center, Tucson, Arizona; g Saranas, Inc, Houston, Texas

**Keywords:** bleeding, high-risk PCI, mechanical circulatory support

## Abstract

**Background:**

High-risk percutaneous coronary intervention (PCI) with mechanical circulatory support (MCS) has been associated with varying rates of bleeding due to variable bleeding definitions, incomplete data relative to site-specific bleeding, and inclusion of variable patient populations.

**Study Design and Objectives:**

SAFE-MCS (NCT05077657) is a multicenter, single-arm, open-label study designed to evaluate the safety of complex high-risk PCI using Impella and surveillance with the Saranas Early Bird Bleed Monitoring System (EBBMS). The study aims to enroll 184 evaluable subjects at up to 15 US centers. The primary clinical end point is the incidence of access-site related BARC type III or V bleeding. Secondary clinical end points include the incidence of each of the Saranas EBBMS level 1, 2, and 3 indicators and the incidence of all BARC type III or V bleeding. Enrollment is anticipated to complete in September 2023 with no longitudinal follow-up.

**Conclusions:**

SAFE-MCS is the first study to exclusively assess bleeding complications in patients undergoing PCI with Impella with independent adjudication via a clinical end point committee and will gather meaningful real-world data using contemporary practice.

## Highlights

• Bleeding is frequent during high-risk percutaneous coronary intervention (PCI) assisted with Impella.
• SAFE-MCS study the impact of Saranas Early Bird Bleed Monitoring System during PCI with Impella.
• SAFE-MCS will enroll 184 patients at up to 15 US centers.
• The primary end point is the incidence of access-site related BARC type III or V bleeding.

## Central Illustration

**Figure:**
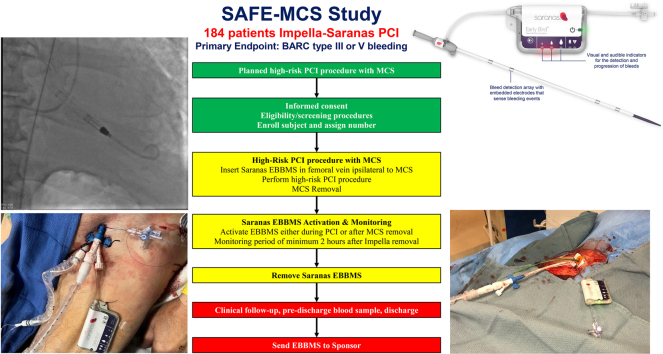

Periprocedural bleeding is the most common complication in patients undergoing percutaneous coronary intervention (PCI) and is associated with a significantly increased risk of mortality, morbidity, and cost.^1^ Recently, the acuity and complexity of PCI have increased, and the use of mechanical circulatory support (MCS) has expanded,^2^ leading to an inherent increase in procedural and bleeding complications.^3^, ^4^, ^5^, ^6^, ^7^, ^8^ Despite the development and implementation of safe femoral access techniques (including the use of micropuncture needles and direct visualization using ultrasound and fluoroscopy)^9^^,^^10^ as well as adjunctive techniques,^11^ vascular and bleeding complications remain frequent, especially when large-bore devices are used.^1^

Early detection of access-related bleeding complications remains challenging, as clinical recognition mainly relies on the occurrence of signs and symptoms (hematoma, pain, hypotension, and sometimes death) and additional imaging confirmation (ultrasound, computed tomography), which usually occur when bleeding is clinically severe. Early awareness of internal bleeding allows physicians to directly and immediately address this complication and increase postprocedural vigilance in these vascular interventions. The Early Bird Bleed Monitoring System (EBBMS; Saranas) is a novel technology that has been designed to detect the onset and progression of an internal bleed and to provide early notification to clinicians.^12^^,^^13^

## The Saranas Early Bird Bleed Monitoring System

**Figure 1:**
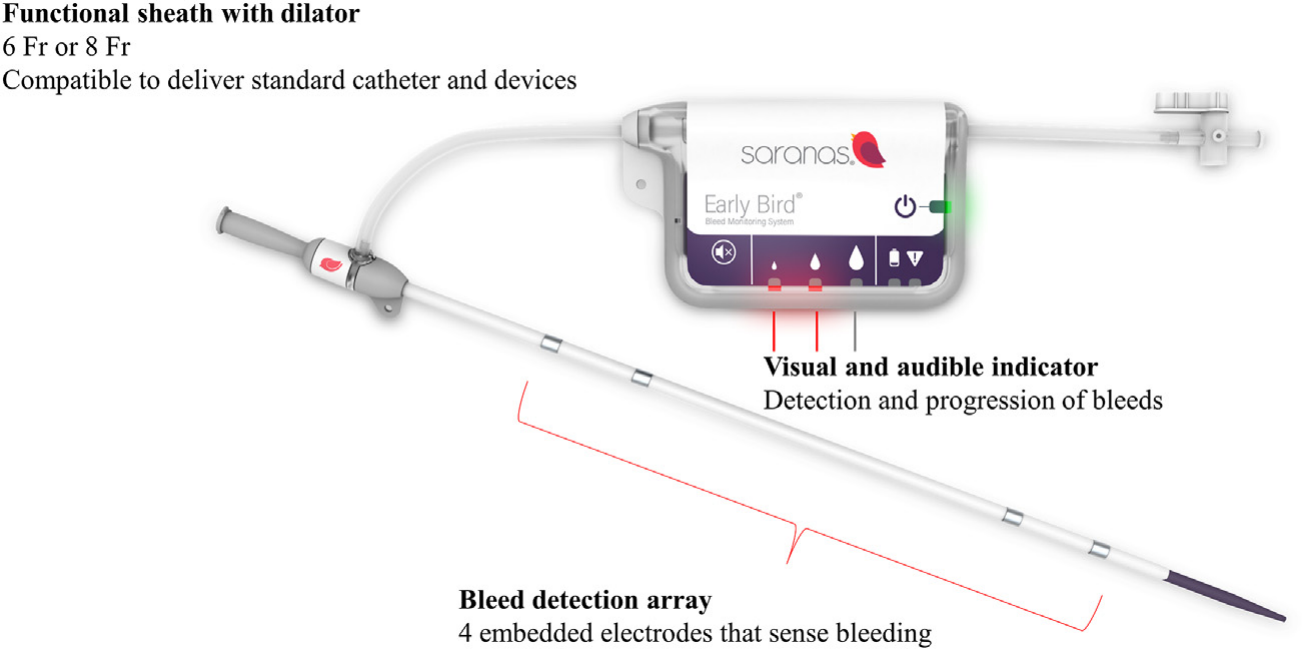
**The Early Bird Bleed Monitoring System from Saranas.** The system consists of a standard 6F or 8F endovascular access sheath with embedded electrodes and a user interface display, which houses a printed circuit board assembly running an algorithm that analyzes bioimpedance and can trigger visible and audible indicators to communicate the state of change in bioimpedance.

The Saranas EBBMS is a real-time bleed detection system that continuously senses and measures potential changes in regional bioimpedance, which indicates a potential bleeding event. As a fully functional introducer sheath, the EBBMS consists of (1) a vascular access sheath with 4 embedded electrodes positioned along the length of the cannula that form a bioimpedance measurement circuit and (2) a user interface display that houses a printed circuit board assembly running an algorithm that analyzes bioimpedance, which can trigger visible and audible indicators to communicate the state of change in bioimpedance to the physician, as depicted by the larger blood drop symbols (Figure 1).

**Figure 2:**
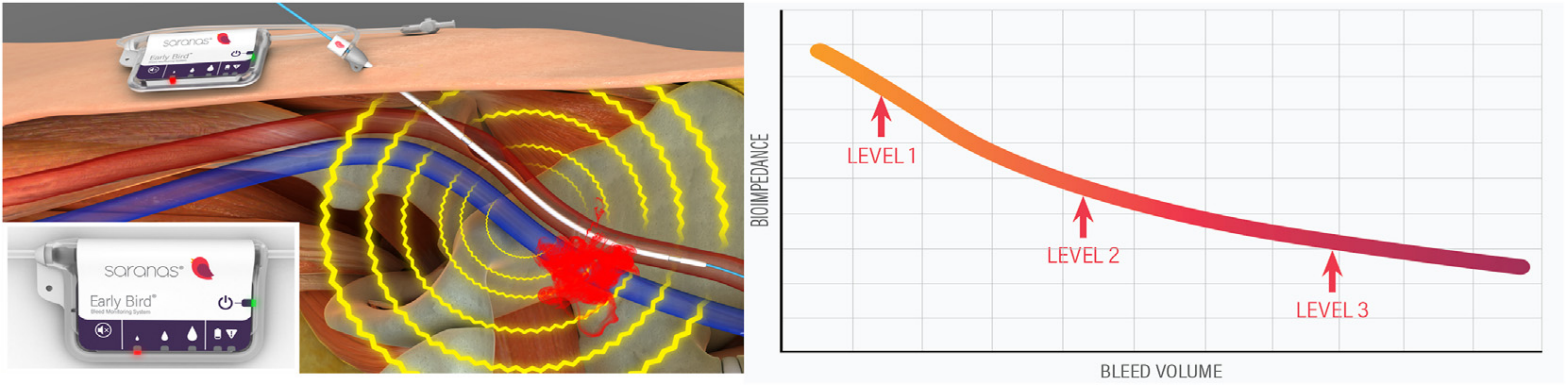
**The Early Bird Bleed Monitoring System and relationship between bioimpedance and bleed monitoring.** The Early Bird Bleed Monitoring System provides a bleed detection array via embedded electrodes, continuously monitoring and interrogating changes in regional bioimpedance. It has been demonstrated that there is a consistent correlation between a decrease in bioimpedance and an increase in extravascular fluid accumulation (bleeding).^12^

After Saranas EBBMS insertion and activation, baseline bioimpedance readings are collected, and the device detects changes in bioimpedance as a response to an active bleed (Figure 2). The user interface display features a 3-level bleed indicator system that sequentially illuminates light emitting diode (LED) indicators to show an increase in bleed progression at the following levels:

• Level 1 indicator (first LED) is triggered by the early onset of a bleed. An audible alert is momentarily activated once this level is triggered.
• Level 2 indicator (second LED) is triggered as the bleed progresses when a bioimpedance threshold is reached. An audible alert, longer in duration than the first LED, is momentarily activated once this level is triggered.
• Level 3 indicator (third LED) is triggered as the bleed continues to progress further when a higher bioimpedance threshold is reached. An audible alert is activated once this level is triggered and requires the attending clinician to silence the device by pressing the silence button.

Prior work in an animal model has demonstrated the performance of the EBBMS (sensitivity and specificity of 100%) in detecting the onset of access-related bleeds as low as ∼50 mL (level 1) and to continuously assess its progression (level 2, ∼100 mL; level 3, ∼200 mL).^12^ A first-in-human study of patients (N = 60) undergoing a variety of elective endovascular procedures demonstrated the safety and accuracy of the EBBMS for the real-time detection and monitoring of access site-related bleeding events. The study demonstrated a high level of agreement between the EBBMS and postprocedural computed tomography to identify periprocedural bleeding events. Indeed, the EBBMS correctly identified bleeding status (bleed or no bleed) in all patients, which included 35% of patients with no bleed and 65% with a bleed, as confirmed by computed tomography. Importantly, among patients with EBBMS-reported bleeding, approximately one-third occurred during the procedure and approximately two-thirds occurred after the procedure.^13^

## Design of the SAFE-MCS study

The Safe Surveillance of PCI Under Mechanical Circulatory Support with the Saranas Early Bird Bleed Monitoring System (SAFE-MCS; NCT05077657) is a multicenter, single-arm, open-label study to evaluate the safety of complex high-risk PCI using the Impella (Abiomed) in conjunction with the Saranas EBBMS. The study will enroll 184 evaluable subjects at up to 15 US centers. Evaluable subjects are defined as all participants who had an EBBMS placed ipsilateral to MCS and had postprocedural monitoring by the EBBMS for a minimum of 2 hours. The study is anticipated to be approximately 24 months in duration. The duration of each subject’s participation will be the duration of the index hospitalization. The study is funded by Saranas, Inc, with an independent clinical events adjudication committee (Cardiovascular Research Foundation) and an independent imaging core laboratory (Medical Metrics).

### Study population

The complete inclusion and exclusion criteria are shown in Table 1. Briefly, patients who are ≥18 years of age who plan to undergo a complex and high-risk PCI requiring MCS with an Impella 2.5 or CP (Abiomed) via transfemoral arterial are eligible for the study. The Saranas EBBMS will be used in the ipsilateral femoral vein to monitor bleeding events during the periprocedural period. The study subject will be informed of the nature of the study, agree to its provisions, and provide written informed consent as approved by the institutional review board of the respective clinical site. Key exclusion criteria include absolute contraindications or allergy to iodinated contrast that cannot be adequately treated with premedication; active bleeding; incapacity to safely access the femoral artery or femoral vein; significant femoral, iliac, abdominal, or thoracic aortic disease that precludes placement of MCS; anemia (hemoglobin <9 g/dL), thrombocytopenia (platelet count <50,000 cells/mL), history of bleeding diathesis or coagulopathy, or hypercoagulable states; active infection not controlled with antibiotic therapy; current pregnancy or women of child-bearing potential without documented negative pregnancy test; estimated life expectancy <24 hours; and patient is in cardiogenic shock at the time of enrollment.

**Table 1:** SAFE-MCS inclusion and exclusion criteria.

**Inclusion criteria**
Patients must meet the following inclusion criteria to be eligible for the study. • ≥18 y of age • Planned complex high-risk PCI using MCS with Impella from a femoral access and use of Saranas Early Bird Bleed Monitoring System • The study patient has been informed of the nature of the study, agrees to its provisions, and has provided written informed consent as approved by the institutional review board of the respective clinical site.
**Exclusion criteria**
Patients meeting any of the following criteria will be excluded from the study. • Absolute contraindications or allergy to iodinated contrast that cannot be adequately treated with premedication • Active bleeding • Incapacity to safely access femoral artery or femoral vein • Significant femoral, iliac, abdominal, or thoracic aortic disease that precludes placement of MCS • Anemia (hemoglobin <9 g/dL), thrombocytopenia (platelet count <50,000 cells/mL), history of bleeding diathesis or coagulopathy, or hypercoagulable states • Active infection not controlled with antibiotic therapy • Currently pregnant or women of child-bearing potential without documented negative pregnancy test • Estimated life expectancy <24 h • Patient is in cardiogenic shock at the time of enrollment.

MCS, mechanical circulatory support; PCI, percutaneous coronary intervention.

Withdrawal of a subject from the study will be at the investigator’s discretion with consideration of the safety and well-being of the subject. Subjects may withdraw from the study at any point.

### Primary end points

The primary end point is incidence of access-site related bleeding (Bleeding Academic Research Consortium [BARC]^14^ type III or V). For the principal analysis of the primary end point, bleeding events are adjudicated per the BARC bleeding criteria shown in Table 2.

**Table 2:** BARC bleeding criteria.

**Type 0:** No bleeding
**Type 1:** Bleeding that is not actionable and does not cause the patient to seek unscheduled performance of studies, hospitalization, or treatment by a healthcare professional; may include episodes leading to self-discontinuation of medical therapy by the patient without consulting a healthcare professional
**Type 2:** Any overt, actionable sign of hemorrhage (eg, more bleeding than would be expected for a clinical circumstance, including bleeding found by imaging alone) that does not fit the criteria for type 3, 4, or 5 but does meet at least 1 of the following criteria: (1) requiring nonsurgical, medical intervention by a healthcare professional, (2) leading to hospitalization or increased level of care, or (3) prompting evaluation
**Type 3a:** Overt bleeding plus hemoglobin drop of 3 to 5 g/dL^a^ (provided hemoglobin drop is related to bleed); any transfusion with overt bleeding
**Type 3b:** Overt bleeding plus hemoglobin drop 5 g/dL^a^ (provided hemoglobin drop is related to bleed); cardiac tamponade; bleeding requiring surgical intervention for control (excluding dental/nasal/skin/hemorrhoid); bleeding requiring intravenous vasoactive agents;
**Type 3c:** Intracranial hemorrhage (does not include microbleeds or hemorrhagic transformation, does include intraspinal); subcategories confirmed by autopsy or imaging or lumbar puncture; intraocular bleed compromising vision
**Type 4:** CABG-related bleeding; perioperative intracranial bleeding within 48 h; reoperation after closure of sternotomy for the purpose of controlling bleeding; transfusion of 5 U whole blood or packed red blood cells within a 48-h period^b^; chest tube output 2L within a 24-h period
**Type 5a:** Probable fatal bleeding; no autopsy or imaging confirmation but clinically suspicious
**Type 5b**: Definite fatal bleeding; overt bleeding or autopsy or imaging confirmation

Platelet transfusions should be recorded and reported but are not included in these definitions until further information is obtained about the relationship to outcomes. If a CABG-related bleed is not adjudicated as at least a type 3 severity event, it will be classified as not a bleeding event. If a bleeding event occurs with a clear temporal relationship to CABG (ie, within a 48-h time frame) but does not meet type 4 severity criteria, it will be classified as not a bleeding event.

BARC, Bleeding Academic Research Consortium; CABG, coronary artery bypass graft.

a Corrected for transfusion (1 U packed red blood cells or 1 U whole blood 1 g/dL hemoglobin).

b Cell saver products are not counted.

### Secondary end points

The secondary end points are the incidence of each of the Saranas EBBMS level 1, 2, and 3 indicators and the incidence of all BARC type III or V bleeding.

### Safety end points

The following end points will be analyzed, as applicable: access site-related bleeding complications; access site-related vascular complications; access site-related blood transfusions; non–access site-related bleeding complications; non–access site-related vascular complications; non–access site-related blood transfusions; all blood transfusions; hemoglobin drop; death; device and procedure-related adverse events; serious adverse events; and serious adverse device effects. All safety end points will be adjudicated per the BARC bleeding criteria (Table 2), the Valve Academic Research Consortium 3 (VARC-3)^15^ vascular and access-related complications definitions (Supplemental Appendix Table 1), and the VARC-3 bleeding and transfusions criteria (Supplemental Appendix Table 2).

### Health economics

In addition to the main clinical study, data from the SAFE-MCS study will be used to perform an economic analysis of the potential economic benefit of the Saranas EBBMS. Since the SAFE-MCS study lacks a concurrent control group, external data will be used to estimate the frequency and costs of bleeding complications in patients treated according to standard of care. Costs associated with bleeding and vascular complications of large-bore access will be estimated using data from either the Nationwide Inpatient Sample or National Readmission Database. Then, based on historical data, a decision-analytic model will be developed to estimate the cost offsets associated with the Saranas EBBMS.

To support the health economics effort of the study, each study site will perform a retrospective chart review of the 10 most recent complex high-risk PCI cases with Impella support without the use of the Saranas EBBMS (∼150 patients) that meet the inclusion and exclusion criteria for SAFE-MCS. These data will be used to estimate the frequency of specific bleeding and vascular complications in patients who underwent Impella-supported PCI without EBBMS monitoring prior to the start of the SAFE-MCS study. Resources utilization, cost, and outcomes will be compared between the 2 groups retrospectively.

Because historical data may overestimate the complication rate in contemporary practice, sensitivity analyses will be performed to examine the relationship between the expected rate of complications with standard practice and the projected cost savings with the Saranas EBBMS.

### Study procedure

**Figure 3:**
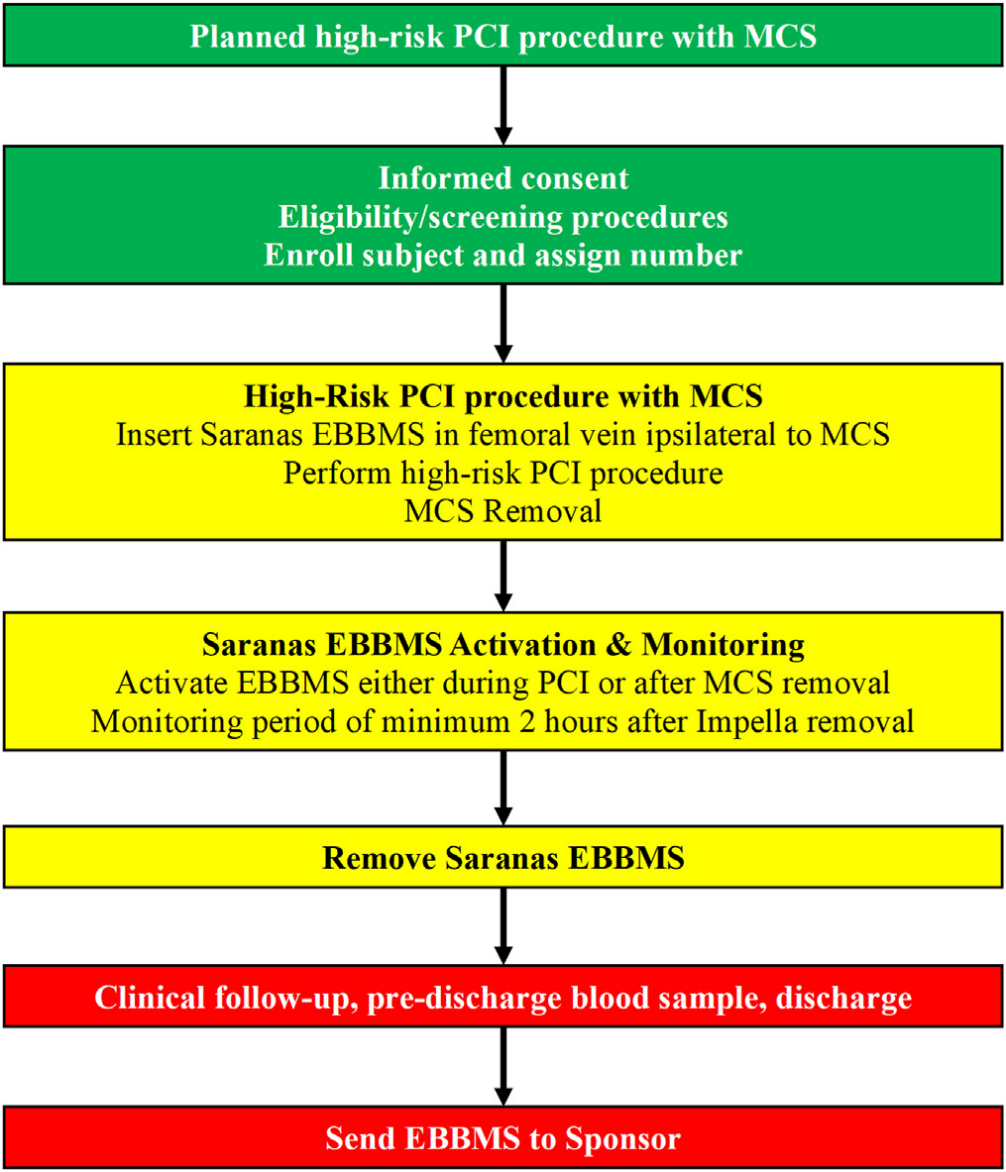
**Study flowchart of the SAFE-MCS study.** The study is divided into 3 timepoints: preprocedure (green), day of procedure (yellow), and postprocedure (red). EBBMS, Early Bird Bleed Monitoring System; MCS, mechanical circulatory support; PCI, percutaneous coronary intervention.

The study is divided into 3 timepoints: preprocedure, day of procedure, and postprocedure. Figure 3 summarizes the study flowchart. During the preprocedure timepoint, the Impella endovascular access procedure will be planned, and an assessment of baseline signs and symptoms will be collected. On the day of the procedure, high-risk PCI with Impella will be performed in accordance with the institution’s standard practice. The Saranas EBBMS sheath will be inserted in the femoral vein, ipsilateral to the Impella device (Central Illustration). Briefly, the data to be collected are the type of coronary intervention under Impella support; type of Impella device; type of Impella sheath; Impella bore sheath size; type of vessel cannulated with the Saranas EBBMS (eg, femoral vein); access technique (eg, ultrasound-guided, micropuncture, anterior vs posterior puncture); investigator’s actions in response to any bleed detection, eg, anti-thrombotic reversal, termination of the procedure, additional imaging, or interventions; and recording of adverse events and/or device complications. If angiograms are performed before and after the large-bore arterial access, these will be sent to the imaging core laboratory for adjudication of any bleeding or safety events.

**Central Illustration:**
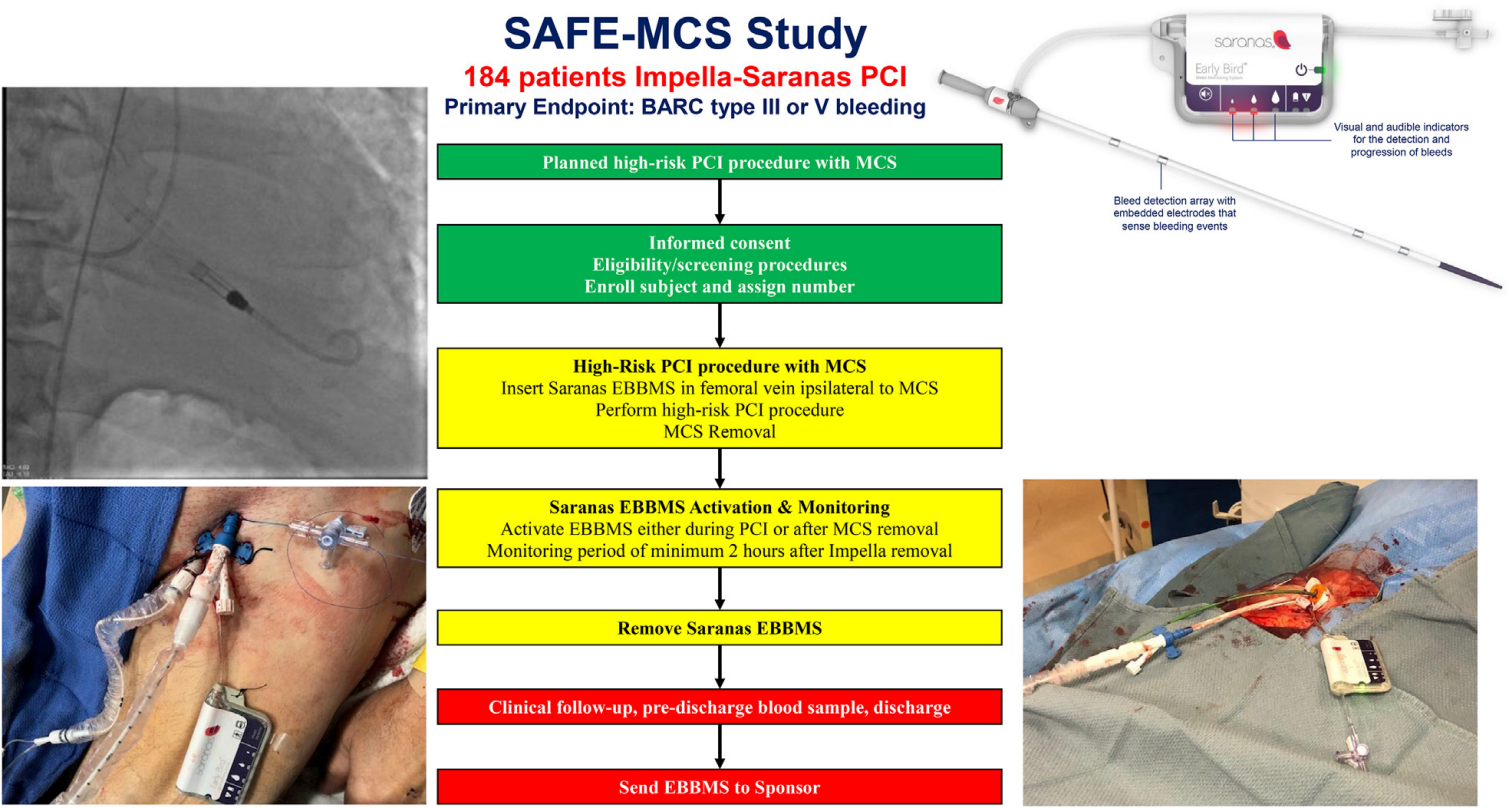
The SAFE-MCS study will enroll 184 patients undergoing complex PCI supported by Impella with the use of a Saranas Early Bird Bleed Monitoring System in the ipsilateral femoral vein. The primary endpoint is the in-hospital rate of access-site related bleeding BARC III or V**.** EBBMS, Early Bird Bleed Monitoring System; MCS, mechanical circulatory support; PCI, percutaneous coronary intervention; SAFE-MCS, Safe Surveillance of PCI Under Mechanical Circulatory Support with the Saranas Early Bird Bleed Monitoring System.

Either during the PCI procedure or after removal of the Impella and closure of the arterial entry site, the Saranas EBBMS will be activated and remain in the femoral vein to monitor for a minimum of 2 hours postprocedure. Once the device is removed and the subject is discharged, the device will be shipped for decontamination and assessment of the impedance response by the sponsor.

## Statistical considerations

### Sample size calculations and assumptions

The primary efficacy aim of this study is to demonstrate the incidence rate of access site-related BARC type III or V bleeding is reduced compared with the historical incidence rate. The secondary efficacy aims are to estimate the incidence rate of activation of each of the EBBMS level 1, 2, and 3 indicators and the incidence rate of all BARC type III or V bleeding and to investigate whether there is an association between the level of indicator and BARC type. The safety aim is to estimate the overall complication/adverse event rate as well as the adverse event rates related to the EBBMS.

The PROTECT II study enrolled 216 patients undergoing nonemergent high-risk PCI under MCS with Impella support.^16^ Investigators reported a major bleeding rate (defined as BARC III or V bleeding) of 12.5%. In the SAFE-MCS study, a 50% reduction in access-site bleeding rate (P) under surveillance with the EBBMS is estimated. The magnitude of reduction was chosen based on what was perceived to be clinically meaningful by the medical trial leadership and took into account that not all bleeding events are access-related and could be reduced by the use of the Saranas EBBMS. Assuming that the rate of bleeding (P) is 6.25%, a sample size of 184 evaluable subjects is required to achieve 90% power for a 1-sample exact binomial test at the 1-sided 0.05 alpha level.

Data will be summarized by sample size (N), mean, standard deviation, minimum, median, and maximum for continuous variables and by sample size and frequency and percentage of discrete variables. For all subjects included in this study, demographics (age, sex, race/ethnicity), medical history, physical examination, and laboratory tests will be summarized.

### Analysis of primary efficacy end points

The incidence of access-related BARC type III or V bleeding events will be defined dichotomously as bleed or no bleed. The frequency and proportion of subjects with a primary efficacy end point will be calculated along with the upper 95% confidence bound. Success will be based on showing the confidence bound is less than the historical rate of 12.5%

### Analysis of secondary efficacy end points

The frequency and percentage of activation of each EBBMS indicator (none/level 1/level 2/level 3) and all BARC type III or V bleeding (no bleeding/type III/type V) will be assessed.

### Assessment of safety

Safety will be assessed from device- and procedure-related adverse events, serious adverse events, and serious adverse device effects. The rate of each type of safety outcome as well as overall safety events will be computed for the study population. Safety and tolerability will be assessed by evaluating reported adverse events as well as any postprocedural changes in values from baseline of clinical laboratory assessments. The incidence of treatment-emergent adverse events will be reported overall and by severity and relatedness to study intervention. Adverse events causing study discontinuation and serious adverse events will be listed and tabulated.

### Limitations

Although the SAFE-MCS study is the first study dedicated to evaluate bleeding events post-Impella use, some limitations exist. First, the current study is a single-arm study, with no randomization to patients undergoing high-risk PCI with Impella support with no Saranas EBBMS. Second, the sample size is modest. Third, patients in cardiogenic shock or ST-elevation myocardial infarction were excluded. Those patients are known to have high bleeding risk and could have enriched our study population. Future studies will target specifically those patients.

## References

[1] Redfors B., Watson B.M., McAndrew T. (2017). Mortality, length of stay, and cost implications of procedural bleeding after percutaneous interventions using large-bore catheters. JAMA Cardiol.

[2] Amin A.P., Spertus J.A., Curtis J.P. (2020). The evolving landscape of Impella use in the United States among patients undergoing percutaneous coronary intervention with mechanical circulatory support. Circulation.

[3] Dhruva S.S., Ross J.S., Mortazavi B.J. (2020). Association of use of an intravascular microaxial left ventricular assist device vs intra-aortic balloon pump with in-hospital mortality and major bleeding among patients with acute myocardial infarction complicated by cardiogenic shock. JAMA.

[4] Kuno T., Takagi H., Ando T. (2021). Safety and efficacy of mechanical circulatory support with Impella or intra-aortic balloon pump for high-risk percutaneous coronary intervention and/or cardiogenic shock: insights from a network meta-analysis of randomized trials. Catheter Cardiovasc Interv.

[5] Pahuja M., Ranka S., Chehab O. (2021). Incidence and clinical outcomes of bleeding complications and acute limb ischemia in STEMI and cardiogenic shock. Catheter Cardiovasc Interv.

[6] Lansky A.J., Tirziu D., Moses J.W. (2022). Impella versus intra-aortic balloon pump for high-risk PCI: a propensity-adjusted large-scale claims dataset analysis. Am J Cardiol.

[7] Pahuja M., Johnson A., Kabir R. (2022). Randomized trials of percutaneous microaxial flow pump devices: JACC state-of-the-art review. J Am Coll Cardiol.

[8] Zeitouni M., Marquis-Gravel G., Smilowitz N.R. (2022). Prophylactic mechanical circulatory support use in elective percutaneous coronary intervention for patients with stable coronary artery disease. Circ Cardiovasc Interv.

[9] Seto A.H., Abu-Fadel M.S., Sparling J.M. (2010). Real-time ultrasound guidance facilitates femoral arterial access and reduces vascular complications: FAUST (Femoral Arterial Access With Ultrasound Trial). JACC Cardiovasc Interv.

[10] Jolly S.S., AlRashidi S., d’Entremont M.A. (2022). Routine ultrasonography guidance for femoral vascular access for cardiac procedures: the UNIVERSAL randomized clinical trial. JAMA Cardiol.

[11] Genereux P., Kodali S., Leon M.B. (2011). Clinical outcomes using a new crossover balloon occlusion technique for percutaneous closure after transfemoral aortic valve implantation. JACC Cardiovasc Interv.

[12] Généreux P., Bueche K., Vondran J., Chuang A., Razavi M. (2020). Safety and accuracy of a novel bioimpedance system for real-time detection and monitoring of endovascular procedure-related bleeding in a porcine model. J Invasive Cardiol.

[13] Généreux P., Nazif T.M., George J.K. (2020). First-in-human study of the Saranas Early Bird Bleed Monitoring System for the detection of endovascular procedure-related bleeding events. J Invasive Cardiol.

[14] Mehran R., Rao S.V., Bhatt D.L. (2011). Standardized bleeding definitions for cardiovascular clinical trials: a consensus report from the Bleeding Academic Research Consortium. Circulation.

[15] Généreux P., Piazza N., VARC-3 Writing Committee (2021). Valve Academic Research Consortium 3: updated endpoint definitions for aortic valve clinical research. J Am Coll Cardiol.

[16] O’Neill W.W., Kleiman N.S., Moses J. (2012). A prospective, randomized clinical trial of hemodynamic support with Impella 2.5 versus intra-aortic balloon pump in patients undergoing high-risk percutaneous coronary intervention: the PROTECT II study. Circulation.

