# Supplementary material for: Design and Rationale of the Safe Surveillance of PCI Under Mechanical Circulatory Support With the Saranas Early Bird Bleed Monitoring System (SAFE-MCS) Study
Source: J Soc Cardiovasc Angiogr Interv. 2023 Jul 5;2(5):101049. doi: 10.1016/j.jscai.2023.101049 (PMC11308036; doi:10.1016/j.jscai.2023.101049)
Supplement: Supplemental Appendix Table 1 and 2 [file mmc1.docx]

**Supplemental Material**

**Design and Rationale of the Safe Surveillance of PCI Under Mechanical Circulatory Support with the Saranas Early Bird Bleed Monitoring System (SAFE-MCS) Study**

**Running Title:** The SAFE-MCS Study Design

Philippe Généreux, MD^1^, Amir Kaki, MD^2^, Mostafa Naguib, MD^1^, MD, Brittany Fuller, MD^3^, Hursh Naik, MD^4^, Michael Kim, MD^5^, Amirali Masoumi, MD^1^, Thomas Waggoner, DO^6^, Zaffer Syed, MS, MBA^7^, Julia Walsh, MCTM^7^, Dimitri Karmpaliotis, MD, PhD^1^, Mir Babar Basir, DO^3^

^1^Gagnon Cardiovascular Institute, Morristown Medical Center, Morristown, NJ; ^2^ Ascension St. John Hospital, Detroit, MI; ^3^Henry Ford Health System, Detroit, MI; ^4^St. Joseph's Medical Center, Phoenix, AZ; ^5^Lenox Hill Hospital/Northwell Health, New York, NY; ^6^ USHV Heart & Vascular/Tucson Medical Center/Tucson, Arizona ^7^Saranas, Inc., Houston, TX

**Table 1. VARC-3^1^ Vascular and Access-Related Complications**

| Vascular Complications^†^ |
| --- |
| Major  One of the following:   - Aortic dissection or aortic rupture - Vascular (arterial or venous) injury (perforation, rupture, dissection, stenosis, ischemia, arterial or venous thrombosis including pulmonary embolism, arteriovenous fistula, pseudoaneurysm, hematoma, retroperitoneal hematoma, infection) or compartment syndrome resulting in death, VARC type ≥ 2 bleeding, limb or visceral ischemia, or irreversible neurologic impairment - Distal embolization (non-cerebral) from a vascular source resulting in death, amputation, limb or visceral ischemia, or irreversible end-organ damage - Unplanned endovascular or surgical intervention resulting in death, VARC type ≥ 2 bleeding, limb or visceral ischemia, or irreversible neurologic impairment - Closure device failure^‡^ resulting in death, VARC type ≥ 2 bleeding, limb or visceral ischemia, or irreversible neurologic impairment |
| Minor  One of the following:   - Vascular (arterial or venous) injury (perforation, rupture, dissection, stenosis, ischemia, arterial or venous thrombosis including pulmonary embolism, arteriovenous fistula, pseudoaneurysm, hematoma, retroperitoneal hematoma, infection) not resulting in death, VARC type ≥ 2 bleeding, limb or visceral ischemia, or irreversible neurologic impairment - Distal embolization treated with embolectomy and/or thrombectomy, not resulting in death, amputation, limb or visceral ischemia, or irreversible end-organ damage - Any unplanned endovascular or surgical intervention, ultra-sound guided compression, or thrombin injection, not resulting in death, VARC type ≥ 2 bleeding, limb or visceral ischemia, or irreversible neurologic impairment - Closure device failure^‡^ not resulting in death, VARC type ≥ 2 bleeding, limb or visceral ischemia, or irreversible neurologic impairment |
| Access-Related Non-Vascular Complications |
| Major  One of the following:   - Non-vascular structure, non-cardiac structure§ perforation, injury, or infection resulting in death, VARC type ≥ 2 bleeding, irreversible nerve injury or requiring unplanned surgery or percutaneous intervention - Non-vascular access site (e.g., trans-apical left ventricular) perforation, injury, or infection resulting in death, VARC type ≥ 2 bleeding, irreversible nerve injury or requiring unplanned surgery or percutaneous intervention   Minor  One of the following:   - Non-vascular structure, non-cardiac structure^§^ perforation, injury, or infection not resulting in death, VARC type ≥ 2, irreversible nerve injury, or requiring unplanned surgery or percutaneous intervention - Non-vascular access site (e.g., trans-apical left ventricular) perforation, injury, or infection not resulting in death, VARC type ≥ 2 bleeding, irreversible nerve injury or requiring unplanned surgery or percutaneous intervention |

* Any complication related to the device insertion, delivery, and complete removal of all its components (delivery catheter, sheath, guide wire), excluding the actual implantation in the heart

† Any device-related vascular access site and any other accessory access sites (venous or arterial) used during procedure

‡ A failure to achieve hemostasis at the access site, resulting in alternative treatment (other than manual compression or planned adjunctive endovascular balloon inflation)

§ Including, but not limited to, the lung (e.g., pneumothorax), direct nerve injury, access site or wound infection, mediastinitis, sternal instability, wound dehiscence, and inability to close the chest

*From Genereux P et al. J Am Coll Cardiol 2021 Vol. 77 Issue 21 Pages 2717-2746.*

**Table 2. VARC-3 Bleeding and Transfusions**

Overt bleeding^†^ that fulfils one of the following criteria^*^

**Type 1**

- Overt bleeding that does not require surgical or percutaneous intervention, but does require medical intervention by a health care professional, leading to hospitalization, an increased level of care, or medical evaluation (BARC 2)
- Overt bleeding that requires a transfusion of 1 unit of whole blood/red blood cells‡ (BARC 3a)

**Type 2**

- Overt bleeding that requires a transfusion of 2–4 units of whole blood/red blood cells‡ (BARC 3a)
- Overt bleeding associated with a haemoglobin drop of >3 g/dL (>1.86 mmol/L) but <5 g/d (<3.1 mmol/L) (BARC 3a)

**Type 3**

- Overt bleeding in a critical organ, such as intracranial, intraspinal, intraocular, pericardial (associated with haemodynamic compromise/tamponade and necessitating intervention), or intramuscular with compartment syndrome (BARC 3b, BARC 3c)
- Overt bleeding causing hypovolemic shock or severe hypotension (systolic blood pressure <90 mmHg lasting >30 min and not responding to volume resuscitation) or requiring vasopressors or surgery (BARC 3b)
- Overt bleeding requiring reoperation, surgical exploration, or reintervention for the purpose of controlling bleeding (BARC 3b, BARC 4)
- Post-thoracotomy chest tube output **≥**2 L within a 24-h period (BARC 4)
- Overt bleeding requiring a transfusion of **≥**5 units of whole blood/red blood cells (BARC 3a) ‡
- Overt bleeding associated with a haemoglobin drop **≥**5 g/dL (**≥**3.1 mmol/L) (BARC 3b).

**Type 4**

- Overt bleeding leading to death. Should be classified as:
  - Probable: Clinical suspicion (BARC 5a)
  - Definite: Confirmed by autopsy or imaging (BARC 5b)

*The timing, indication, and number of transfused blood products should be collected and reported specifically during the index procedure, during the entire index hospitalization, and during follow-up after discharge, whether or not overt bleeding is identified. †Overt bleeding is defined as any clinically obvious source of bleeding or bleeding source identified after appropriate investigation and diagnostic testing (e.g. imaging). Any procedural blood loss should be considered overt bleeding. ‡Total number of transfusions should be reported separately for (i) within 48 h of the index procedure, (ii) the total duration of the index procedure hospitalization, and (iii) during any subsequent repeat hospitalization

*From Genereux P et al. J Am Coll Cardiol 2021 Vol. 77 Issue 21 Pages 2717-2746*

1. Varc-3 Writing C, Genereux P, Piazza N, Alu MC, Nazif T, Hahn RT, Pibarot P, Bax JJ, Leipsic JA, Blanke P, et al. Valve Academic Research Consortium 3: Updated Endpoint Definitions for Aortic Valve Clinical Research. *J Am Coll Cardiol*. 2021;77:2717-2746. doi: 10.1016/j.jacc.2021.02.038
